# Treatment of Aggressive Behavior and Agitation in an 11-Year-Old Boy with Co-Occurring Autism and ADHD: A Case Report and Literature Review on the Use of Intravenous Valproate in Emergency Psychiatry

**DOI:** 10.3390/jcm13123573

**Published:** 2024-06-18

**Authors:** Alessandra Carta, Vanna Cavassa, Mariangela Valentina Puci, Roberto Averna, Giovanni Sotgiu, Giovanni Valeri, Stefano Vicari, Stefano Sotgiu

**Affiliations:** 1 Child and Adolescent Neuropsychiatry Unit, Department of Medicine, Surgery and Pharmacy, University Hospital of Sassari, Viale San Pietro 43/B, 07100 Sassari, Italy; vanna.cavassa@aouss.it; 2 Department of Biomedical Sciences—Section of Neuroscience and Clinical Pharmacology, University of Cagliari (Branch of Sassari), 09121 Cagliari, Italy; 3 Clinical Epidemiology and Medical Statistics Unit, Department of Medicine, Surgery and Pharmacy, University of Sassari, 07100 Sassari, Italy; mvpuci@uniss.it (M.V.P.); gsotgiu@uniss.it (G.S.); 4 Child and Adolescent Neuropsychiatry Unit, Bambino Gesù Children’s Hospital (OPBG), Scientific Institute for Research, Hospitalization and Healthcare, 00146 Rome, Italy; roberto.averna@opbg.net (R.A.); giovanni.valeri@opbg.net (G.V.); stefano.vicari@opbg.net (S.V.); 5 Life Sciences and Public Health Department, Catholic University, 00168 Rome, Italy

**Keywords:** autism, ADHD, agitation, intravenous valproate

## Abstract

**Background:** Autism spectrum disorder (ASD) is a persistent neurodevelopmental disorder frequently co-occurring with attention-deficit/hyperactivity disorder (ADHD) and behavior-related disorders. While behavioral therapy is the first-line option to manage the core symptoms of ASD, pharmacological therapy is sometimes needed to treat acute problems, such as agitation and aggressive behaviors. Recent guidelines recommend the use of neuroleptics to reduce psychomotor agitation in patients with ASD. However, as children with ASD are often drug-resistant, alternative treatments are often justified. Reports from the literature have indicated that intravenous valproate (IV-VPA) can be effective in reducing agitation in psychiatric patients, with a lower frequency of adverse events compared to conventional treatments. However, as the related findings are occasionally inconsistent, IV-VPA is not yet an approved option in the context of clinical psychiatry. We aim to improve knowledge of the IV-VPA treatment option for emergency psychiatric treatment in pediatric patients. **Methods:** We report the case of an 11-year-old boy suffering from a complex neurodevelopmental condition who experienced a psychotic episode with severe aggressive and disruptive behaviors and was successfully treated with IV-VPA. Furthermore, we provide an updated literature review on this topic. **Conclusion:** In our case, first-line therapies proved to be ineffective. To the contrary, IV-VPA led to safe and prompt clinical success, which is in line with other reports. Based on our literature review, IV-VPA can be highly effective and reduces the risk of adverse events that frequently occur with the use of high-dose standard medications in emergency psychiatry.

## 1. Introduction

Autism spectrum disorder (ASD) is a neurodevelopmental disorder that affects communication, social interaction, and behavioral characteristics, which are defined by restricted interests, repetitive and stereotyped behaviors, and abnormal sensory processing. The clinical presentation of ASD symptoms and their severity can vary greatly, depending on age, cognitive abilities, language abilities, and comorbidities. The most common disorder co-occurring with ASD is attention-deficit hyperactivity/impulsivity disorder (ADHD) [1]. Clinical similarities between the two disorders are common, as they share symptoms of inattention and hyperactivity/impulsivity and a high occurrence of at least one psychiatric condition (approximately 80% in ADHD patients and 75% in ASD patients) [1]. This aspect can complicate the clinical diagnosis [2] and pharmacological management of ASD [3]. It has been described that patients with ASD and ADHD experience episodes of psychomotor agitation, with or without aggressive behaviors, more frequently than the general population compared to children with ASD alone, maybe due to a combination of factors, including cognitive dysfunctions, psychiatric disorders, difficulties in communication, and problems adapting to the surrounding environment [2,3]. This fact is associated with a resistance to conventional treatments, especially pharmacologically [4,5,6,7]. Due to the frequently observed drug resistance in children with autism spectrum disorder (ASD), off-label treatments have been justified [8,9]. In October 2023, the Italian Ministry of Health published recommendations in a guideline on the diagnosis and treatment of ASD in children and adolescents. This guideline suggests that, when clinically necessary, pharmacological interventions should be used to treat comorbidities of ASD (on the following hyperlink: https://www.iss.it/documents/20126/8977108/Linea+Guida+ASD_bambini+e+adolescenti+2023.pdf/e370f693-d569-4490-6d51-8e249cd152b0?t=1696841617387 (accessed on 9 October 2023)) [10].

For this reason, in order to mitigate hyperactivity or psychomotor agitation in people with ASD and ADHD or other associated comorbidities, pharmacological interventions with methylphenidate (MPH) [11,12,13] and second-generation antipsychotics (SGAs) are sometimes necessary to address acute issues such as hyperactivity, agitation, and aggressive symptoms [10,11,12,13].

In children with co-occurring ASD and ADHD, pharmacological interventions may help to improve their participation in conventional behavioral therapies [10] and enhance their daily functioning [12]. However, patients with this condition are also more sensitive to the side effects of medication and are more likely to experience adverse events [13]. Therefore, to avoid this risk, in youth with comorbid ASD–ADHD, pharmacological treatment should be initiated at lower doses and adjusted more slowly than in individuals with a diagnosis of only ADHD [14,15].

As previously explained, these patients with comorbid ASD–ADHD often experience acute psychomotor agitation, defined as a state of heightened anxiety, irritability, increased motor and verbal activities, uncooperativeness, and threatening gestures, more so than patients with only one of the two neurodevelopmental disorders [2,8]. In principle, an effective psychopharmacological intervention should achieve a reduction in agitation, promote calmness, and restore contact within two hours [4]. However, pharmacological treatment for acute agitation and aggressive behaviors can be challenging for clinicians [3,4,5,6,7,8], and this comorbidity can present unique challenges in terms of diagnosis [2], treatment [11,12,13], and overall management [10]. Understanding the interplay between these two neurodevelopmental disorders is crucial for providing comprehensive and effective care for those patients who have both conditions. Research has shown that individuals with comorbid ASD and ADHD may exhibit more severe symptoms, poorer outcomes and may require tailored treatment approaches that consider the complexities of both conditions.

Some findings have suggested that intravenous valproate (IV-VPA) can effectively reduce agitation in psychiatric patients, with a lower occurrence of side effects compared to traditional treatments [16]. However, inconsistencies in research findings have prevented IV-VPA from being approved as a clinical psychiatric option at this time [16,17].

Valproate (2-propylpentanoate, VPA) is available in various forms, including valproic acid, sodium valproate, and semi-sodium formulations [18]. Its mechanism of action involves enhancing inhibitory neurotransmission and modulating voltage-gated ion channels [19]. It affects dopamine, gamma-aminobutyric acid, and glutamate neurotransmission, as well as intracellular signaling. VPA is a commonly used and approved drug for both children and adults as a safe and effective antiepileptic, available for administration through oral [17,18,19] or intravenous (IV) administration routes [20]. Apart from its FDA indications for the treatment of epilepsy, oral VPA has found off-label applications in several psychiatric conditions, such as maniac episodes associated with bipolar disorder, impulsivity, agitation, and aggression [21,22,23,24].

Oral VPA has also been shown to reduce aggressive, repetitive symptoms and irritability in children with autism spectrum disorder compared to a control group [24,25,26].

A recent study has shown that using off-label IV-VPA can be both effective and safe in treating psychomotor agitation in adolescents aged between 13 and 17 years [4,27,28]. Overall, there is currently a gap in the available literature on the effectiveness of intravenous valproate in treating acute and aggressive agitation in children with ASD [29].

Regarding the use of VPA in emergency care for managing symptoms of aggressiveness or agitation in children with the complex comorbidity between the two disorders of ASD and ADHD, we did not find any existing data on its effectiveness, whether administered intravenous or orally.

One reason for studying the intravenous delivery of valproic acid (IV-VPA) is that it can potentially offer a faster and more effective route of administration compared to oral delivery. IV administration bypasses the digestive system, allowing for quicker absorption and onset of action [30]. This can be particularly beneficial in emergency psychiatric situations, where rapid therapeutic effects are needed [27].

Additionally, IV-VPA may have different pharmacokinetic and pharmacodynamic properties compared to oral administration. For instance, IV-VPA may have a higher bioavailability and yield more predictable blood concentrations of valproic acid, which must be maintained within the therapeutic range of 50 to 100 micrograms/milliliters, which can lead to improved therapeutic outcomes and a reduced risk of side effects. Understanding these differences can help optimize dosing regimens and improve patient outcomes [30].

Overall, studying the IV delivery of VPA is important for maximizing the therapeutic potential of this drug and enhancing its utility in emergency clinical practice. Through investigating its pharmacokinetics, pharmacodynamics, and efficacy through intravenous administration, we can gain valuable insights into its optimal use and the potential benefits for patients.

To implement the increasing knowledge concerning IV-VPA as a treatment option in emergency psychiatry in patients with both ASD and the comorbid conditions of ASD and ADHD, we describe a clinical case in light of previous related reports. This patient did not show any improvement in symptoms with first-line medications. Additionally, he began experiencing severe side effects after receiving high doses of olanzapine, haloperidol, and benzodiazepines. This prompted clinicians to consider a second-line and off-label treatment option that could quickly reduce aggressiveness and agitation.

Furthermore, we present the results of a review on the use of IV-VPA in children with this complex condition.

Finally, including both original experimental research and a detailed review of the existing literature, results are presented through a more thorough analysis of this subject matter, thus providing readers with a more complete understanding of the current state of research in the specific field of the use of IV-VPA through the example of an 11-year-old child with a neurodevelopmental disorder and acute symptoms of psychomotor agitation and aggression.

## 2. Methods and Case Report

Focusing on the comorbidity of ASD and ADHD in the context of IV-VPA treatment, this study aims to address the specific challenges and needs of the complex subgroup of patients with comorbid ASD and ADHD.

Investigating the safety and efficacy of IV-VPA in this population can provide valuable insights into its potential benefits and limitations, in terms of managing the symptoms of both disorders.

Specifically, in the present study, we aim to review the available literature, including clinical reports (cases or clinical series), as well as narrative and systematic reviews, on the use of IV-VPA in the treatment of acute agitation in children with complex neurodevelopmental disorders. The relevant literature was obtained from PubMed biomedical and Medline (via PubMed), Psych-info, Web of Science (WOS), and Cochrane databases, which included only English-written, full-text clinical studies, clinical reports, and reviews. With regard to interventional studies, we only included those that provided the ethical approval code. The search began on 18 September 2023, applying the following key terms: “autism” and “ADHD” and “neurodevelopmental disorders” and “aggressive behavior” and “acute agitation” and “valproic acid”.

Although VPA was approved in 1978, the literature prior to 1995 was overlooked in our review due to the historical focus on its use for seizures in earlier years, resulting in fewer studies and publications on the use of VPA specifically for psychiatric conditions. Additionally, another reason is that the standards for conducting and reporting scientific research were not as stringent before the 1990s, which raises concerns regarding the reliability and accuracy of older studies.

**Figure 1:**
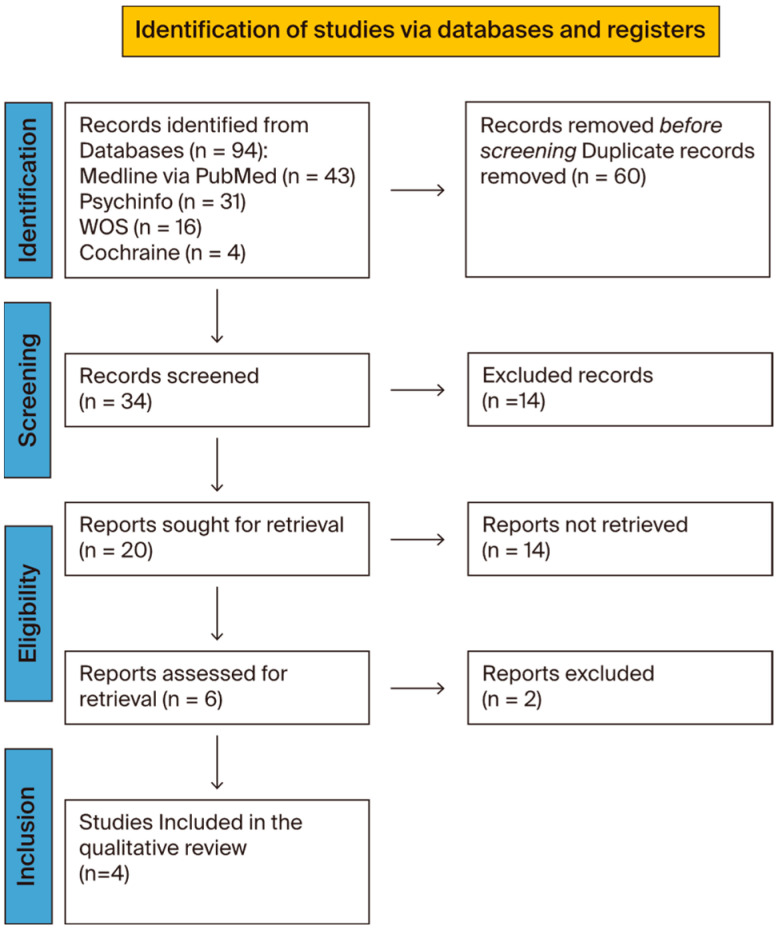
Study selection flowchart. PRISMA 2020 flow diagram for new systematic reviews, which included searches of databases [39]. This figure shows a flowchart detailing the selection criteria for the collected articles.

Therefore, the search period was set between 1995 and 2023. The criteria for inclusion in the screening were individuals receiving VPA therapy for acute agitation and/or aggressive symptoms. Data extraction included a total of 93 articles, and the study selection flow chart—following the Preferred Reporting Items for Systematic Reviews and Meta-Analyses (PRISMA) 2020 flow diagrams—is presented in Figure 1. After removing duplicates, out of a total of 65 articles, 34 were evaluated based on their title and abstract. Of these, 20 articles were deemed eligible based on their keywords and inclusion criteria, which were then evaluated at the full-text level. From the initial selection, we only considered articles in which VPA was administered via the intravenous route. In order to produce a qualitative synthesis on treatment efficacy, we identified 4 studies that met the eligibility criteria regarding patients’ age (children and adolescents) and intravenous administration of VPA. The exclusion criteria were: age over 18 years, oral administration of VPA for chronic use, and not for the treatment of acute agitation with aggressiveness.

To date, to the best of our knowledge, only one report has been published that describes the use of intravenous valproic acid (IV-VPA) for treating acute agitation in children with autism. We did not find any studies evaluating the effectiveness of IV-VPA in the context of complex neurodevelopmental conditions or in patients with both autism spectrum disorder (ASD) and attention-deficit/hyperactivity disorder (ADHD).

Considering that there is limited evidence on the use of IV-VPA in children and adolescents with neurodevelopmental disorders (we only found one case report by Hilthy et al. [29]—especially in cases of acute psychiatric symptoms, aggressive behavior, and psychomotor agitation—we chose to include all study designs in order to summarize all of the available data on the use of IV-VPA in this population. Therefore, all types of diagnoses (including psychosis and mania) were included. Diagnoses should be made according to the Diagnostic and Statistical Manual of Mental Disorders (DSM; American Psychiatric Association, 1980, 2000, 2013) or the International Classification of Diseases (ICD; World Health Organization, 1992). Only studies that reported data on agitation or aggressive behaviors and used a standardized and validated assessment instrument were included. The data regarding the outcome measures were obtained using the following measures: the Aberrant Behavior Checklist (Aman, Singh, Stewart, & Field, 1985), the Brief Psychiatric Rating Scale (Faustman & Overall J, 1999), the Bech–Rafaelsen Mania Scale (Bech, 2002), the Clinical global impression (Jagadheesan, et al., 2003), the Children’s Yale-Brown Obsessive Compulsive Scale (Goodman, et al., 1999), the Mini Mental State Examination (Teng & Chui, 1987), the Overt Aggression Scale (Silver & Yudofsky, 1991), and the Modified Overt Aggression Scale (Kay, Wolkenfeld, & Murrill, 1988) [31,32,33,34,35,36,37,38].

Due to the lack of previous findings described above, in order to enhance the existing literature, we hereby present a case of a young boy diagnosed with a complex neurodevelopmental disorder characterized by the comorbidity between ASD and ADHD.

## 3. Case Report

We describe an 11-year-old boy who had been diagnosed with level 1 autism spectrum disorder (ASD), attention-deficit/hyperactivity disorder (ADHD)—combined presentation, oppositional defiant disorder, and adjustment disorder, according to the DSM-5.

In March 2023, he was re-admitted to our emergency hospital department due to the sudden onset of bizarre behavior (e.g., stripping naked in public), disruptive mood (including verbal opposition and rule-breaking behaviors with everyone, including family members), and severe destructive symptoms (destroying tools and furnishing accessories). His parents noticed a decline in their child’s behavior starting in January 2023, attributing it to unsupervised internet access and contact with unfamiliar adults who had solicited virtual sexual interactions.

### 3.1. Medical History

The patient had a family history of alcohol abuse and socio-economic problems. He was born following a regular pregnancy, delivered naturally at 41 weeks. Parents reported no complications during birth. A delay in language skills was noted, with the first words spoken at 2 years old. The initial difficulties in social abilities were observed during kindergarten, characterized by a deficiency in demonstrating spontaneous interest in others and initiating interactions during peer activities. He also struggled with accepting changes in his routines, such as taking off his diapers, changing into new clothes or shoes, or eating foods other than plain pasta or bread. His free-play was extremely poor, excessively chaotic, and characterized by repetitiveness: rolling objects, throwing them, and going up and down in rigid and afinalistic sequences.

Repetitive patterns of overactivity, delay aversion, and concentration problems were observed during the preschool stage. He was diagnosed with pervasive developmental disorder and received rehabilitation treatments at the local Community Services for Child and Adolescent Neuropsychiatry starting at the age of 3.

In 2019, at the age of 7, he was admitted to the Unit of Child Neuropsychiatry at Sassari Hospital due to an increase in psychomotor agitation and aggressive behaviors towards teachers and peers at school. He exhibited agitation and aggression in response to minor frustrations. During his hospital stay, he displayed abnormalities in social interaction skills, communication deficits, repetitive interests, poor flexibility, intolerance to frustrations, difficulty maintaining eye contact, and challenges in understanding the theory of mind.

The child also showed high levels of hyperactivity and impulsiveness, acting impulsively without thinking and lacking awareness of danger. Several standardized tests were conducted to assess the severity of ADHD symptoms, including the Swanson, Nolan, and Pelham Rating Scale (SNAP-IV) [40], Conners’ Parent Rating Scale [41], and Child Behavior Check List for parents [42], resulting in high scores for both hyperactivity and impulsivity and oppositional defiant disorder (ODD) symptoms. To assess cognitive functioning, we administered the Leiter International Performance Scale, Third Edition [43], revealing fluid reasoning of 77 and a short non-verbal IQ score of 74. He received a diagnosis of severe ADHD with a combined clinical manifestation of severe oppositional and defiant disorder (ODD). Finally, the second version of the Autism Diagnostic Observational Schedule (ADOS-2) [44] confirmed the presence of a co-occurring level 1 autism spectrum disorder (ASD) condition. Genetic analysis (karyotype, FMR1A gene, Pompe disease, Duchenne/Becker disease, Gaucher disease, and Niemann–Pick disease), electroencephalogram, brain MRI, complete abdominal ultrasound, and cardiological examination with echocardiogram all resulted in negative results. The dermatological examination showed six café-au-lait macules, neck and large-fold hyperpigmentation, and follicular hyperkeratosis. At discharge, psycho-stimulant therapy, in addition to cognitive behavioral therapy (CBT) to treat ADHD, was suggested; however, his parents initially refused. After one year, when the child was 8 years old, they came back for a new visit at our outpatient service to evaluate the introduction of methylphenidate (MPH) treatment due to difficulties in managing ADHD symptoms at school and at home. Immediate-release methylphenidate (MPH) was first introduced at a dosage of 0.3 mg/kg. The absence of negative side effects, effective management of inattention symptoms, and a decrease in motor hyperactivity were noted. As a result, the equivalent dosage of prolonged-release MPH was then prescribed to maintain the treatment at home. The child continued with the prescribed therapy, and its effectiveness was reported during periodic checks over the following three years.

### 3.2. Emergency Acceptance

At the emergency room, blood tests (including kidneys, liver, thyroid, and electrolytes), neurological assessment, and urine toxicology tests were all normal. On 2 March 2023, a preliminary electrocardiogram was performed to search for possible contraindications to pharmacological treatment, and it was also normal (see Table 1). Structural heart problems were previously excluded by echocardiogram during a previous hospitalization; furthermore, the heart rate has consistently remained within the age-appropriate range (75–118 beats per minute).

**Figure 2:**
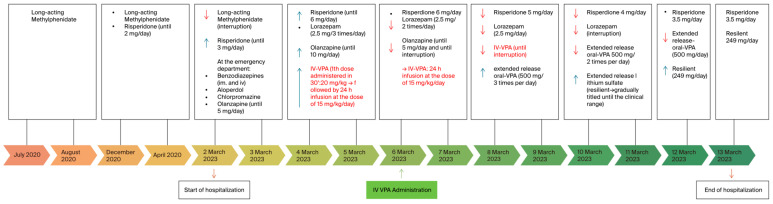
Pharmacological treatment history of the clinical case, showing the timeline of the treatments. In red the IV-VPA administrations. It is important to note a concomitant reduction in the use of other medications after the IV-VPA injection was started. The timeline can also be compared to the MOAS scores presented in Figure 3 below. It is also important to note that, when the IV-VPA treatment started, the other treatments decreased.

After this initial step, the boy was promptly treated with multiple medications at high dosages (haloperidol, benzodiazepines, and chlorpromazine), despite their poor effectiveness in treating aggressive behavior and the quick onset of several side effects, such as sedation, excessive salivation, and rigidity (a detailed pharmacological timeline is shown in Figure 2). MPH was immediately interrupted to avoid a maniac phase induction, at the age of 11 years.

Olanzapine was introduced at the Emergency Department before hospitalization at the Child and Adolescents Unit, as the previous treatment was not effective in managing agitation and aggressive behaviors. It was used at a dosage of 10 mg per day but was gradually stopped after four days due to a lack of positive response. Risperidone was gradually modulated up to 6 mg/day, and extended-release lithium sulfate therapy was introduced to enhance the neuroleptic effect, titrated until achieving a blood concentration within the range of 0.8 mEq/L (see Figure 2). Unfortunately, acute symptoms persisted. The increasing extrapyramidal symptoms observed, such as rigidity and sialorrhea—likely as a side effect of the highest doses of olanzapine added to the risperidone—along with the lack of improvement in agitation and aggression symptoms, were the principal factors that directed clinicians towards a therapeutic change. Therefore, on the third day of hospitalization, we administered an IV bolus of 20 mg/kg of VPA over 30 min. Following that, we initiated a 24-hour infusion at the same dosage, followed by three days of IV infusion at a lower dosage of 15 mg/kg per day. The dosage of IV-VPA was determined in line with previous case descriptions on the use of IV-VPA in adolescents [18], based on the patient’s weight, age, and current medical condition. The healthcare team calculated the appropriate dosage to achieve therapeutic levels of VPA in the patient’s bloodstream (refer to Table 1). To minimize the risk of side effects, vital signs, blood pressure, and heart frequency were also constantly monitored. The decision to initiate IV administration of VPA was made with the rationale of quickly establishing therapeutic levels in the patient’s bloodstream, especially as clinicians observed a deterioration in the severity of the symptoms. Moreover, the patient displayed marked and growing oppositional and non-compliant behaviors towards doctors, nurses, and even their parents, and refused to take medications orally. A successful immediate response was observed shortly after the first few hours, and intravenous valproic acid was switched to the oral formulation. This decision was based on the patient’s response to IV-VPA, based on the easier administration of oral medications as soon as the clinical conditions allowed, and based on the blood levels of ammonia, which had increased beyond the clinical range during the days of the infusion (see Table 1), although there were no clinical signs or symptoms of intoxication.

For all of the abovementioned reasons, on the fourth day, IV-VPA was replaced with its extended oral formulation and gradually decreased over the course of five days (refer to Figure 2), resulting in a positive response and no adverse effects from the medication. Benzodiazepines and olanzapine were also gradually reduced, while treatment with risperidone and lithium was continued.

**Figure 3:**
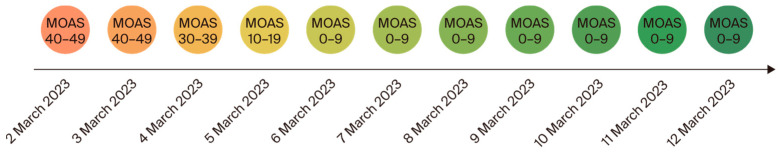
MOAS aggressive behaviors scale. It can be seen, according to the color scale, that there was a rapid reduction in the MOAS scores. The MOAS aggressive behaviors scores showed a reduction on the third day of the hospitalization (on 4 March 2023), immediately after the IV-VPA administration started (compare with Figure 2, showing the pharmacological treatment history of the clinical case, above).

We used the *Modified Version of the Overth Aggression Scale* (MOAS) [38] to assess and measure aggressive behaviors, which showed a decrease from a score of 49 upon admission to a score of 0 upon discharge. Despite mild sedation, an important and rapid decrease in aggressive behaviors occurred after IV-VPA administration, associated with a reduction in the MOAS score (see Figure 3 below).

During hospitalization, electrocardiograms and regular blood tests were conducted to monitor serum levels of VPA, ammonia, lithium, and liver enzymes. The highest VPA value recorded during acute IV treatment was 92.7 μg/mL, which fell within the normal range. The ranges for the other substances are listed in Table 1 above. After 11 days, the patient was discharged with a DSM-5 diagnosis of psychotic onset with psychomotor agitation, along with comorbidities of ASD level 1, severe ADHD combined clinical presentation, and adjustment disorder with mixed emotionality and conduct symptoms. The patient was prescribed home therapy with long-acting lithium sulfate (249 mg/day) and risperidone (3.5 mg/day). It is worth noting that VPA was used during agitated states and later replaced with another stabilizer: lithium sulfate.

### 3.3. One-Year Follow-Up

The patient regularly attended all of the scheduled outpatient visits at our facility. He returned to school just two weeks after being discharged from the hospital and maintained twice-weekly psychotherapy sessions. In October 2023, he started experiencing occasional episodes of agitation and displayed an irritable mood with anger and frustration, particularly when denied food. He exhibited a short temper and would lash out at people in various situations, including classmates, teachers, doctors, and caregivers. His lithium blood level had decreased to 0.45 mEq/L from 0.66 mEq/L a month prior. Therefore, the dosage of long-acting lithium sulfate was increased. A good clinical response, characterized by the absence of new episodes of agitation, aggression, and mood stability, was reported in some weeks.

The use of intravenous valproic acid definitely helped in managing the observed acute aggressive behaviors, as shown by direct assessments using MOAS (Figure 3). However, it is important to note that IV-VPA had no effect on symptoms related to his underlying neurodevelopmental disorders, such as ASD and ADHD, in the long-term follow-up. During the acute phase, he would not have been able to participate in any of the activities that were subsequently planned for him and his family. Prior to discharge, clinicians supported him in starting socio-educational programs to enhance his social skills in various natural-life settings, including home, school, and leisure time with peers. After his return home, the child was also enrolled in a soccer team that he now regularly attends with his personal educator. At present, he is attending the first year of middle school with support teachers and educators for the hours required by Italian law. He benefits from an individualized educational project in the classroom setting, and has achieved learning objectives with a personalized teaching plan. The class group is inclusive towards him, even though his behavior may sometimes be incomprehensible from a social perspective. His reference educator has promoted small group work to explain the child’s behavioral characteristics. Regarding ADHD, his attention span is gradually increasing due to targeted psychotherapy and possibly also to methylphenidate treatment, which has been reintroduced at the minimum effective dose with once-per-day administration in order to help him sustain participation in activities with extended attentive times and lower levels of hyperactivity symptoms.

At the last examination in March 2024, his pharmacological therapy was significantly stable. His parents received support benefits from social assistance and participated in a specific parent program to enhance their educational competencies. This young boy still visits our outpatient service at the institution.

His clinical history and trajectory indicate a high risk of developing additional mood or psychotic episodes, particularly in response to further environmental stressors.

## 4. Results of the Literature Review

A total of 34 articles evaluating the effectiveness of both oral and IV valproic acid (VPA) in various clinical psychiatric settings for children, adolescents, and adults were reviewed based on their titles and abstracts. Among these articles, there were 3 (8.8%) case reports, 1 (2.9%) cohort study, 1 (2.9%) case-control study, 3 (8.8%) clinical trials, 4 (11.7%) meta-analyses, 7 (20.6%) systematic reviews, 12 (35.3%) literature reviews, 3 (8.8%) randomized clinical trial*s*, and 1 (2.9%) thematic analysis (refer to Table 2).

Regarding the administration of VPA, 9 participants (26.4%) received IV-VPA, while 22 (64.7%) received oral VPA. However, the method of administration was not specified in three studies (8.8%). All participants included in the studies exhibited symptoms of acute agitation and aggressiveness, sometimes during manic phases.

In order to better classify acute agitation with aggression, we identified four categorical clinical diagnoses that preceded the acute psychiatric phase. The most frequent diagnosis associated with aggressive agitation was mood disorders (MDs)—particularly bipolar disorder (17 reports)—followed by schizophrenia spectrum disorders (SSDs; 15 reports), including schizophrenia (8 reports) and schizoaffective disorder (7 reports). We also found disruptive behavior disorders (DBDs) in six case descriptions: three cases had conduct disorder, and three other cases had oppositional and defiant disorder. Finally, we retrieved five articles describing trauma and stressor-related disorders (TSDs), with clinical descriptions of post-traumatic stress *(n =* 4) and adjustment disorder (*n =* 1).

Regarding comorbidity, the co-occurrence of MD and acute agitation was more frequently reported when a diagnosis of neurodevelopmental disorders occurred, particularly ADHD and ASD, sometimes in association with anxiety symptoms. Acute agitation in SSD was reported alone—or, in some cases, in association with stressful events—in people with a long clinical history. In nine articles, DBD was reported in patients with a previous diagnosis of ADHD and, in four of them, in association with substance abuse disorder. TSD is usually described as a trigger factor in patients with neurodevelopmental, mood, and/or anxiety disorders.

To the best of our ability, we did not find any article analyzing the specific use of IV-VPA in youth with the complex neurodevelopmental condition represented by co-occurring clinical manifestations of ASD and ADHD.

With regard to treatment efficacy, we selected 20 articles at a full-text level that were in line with the inclusion criteria. Of these, 14 reports were not retrieved.

In detail, these 14 studies were not retrieved as they were considered beyond the scope of our review, which aimed to focus attention on the use of IV-VPA in children and adolescents. In particular, 6 out of 14 of these studies were excluded because they did not include patients under 18 years of age; 3 were excluded because they did not specifically address the use of IV-VPA in acute aggressive behaviors; and, finally, 5 studies were excluded as they did not employ any standardized outcome measures.

Two additional studies were excluded because they involved adults. Finally, only four studies were considered eligible for a literature review: one systematic review, two literature reviews, and two case reports. The articles were coded based on the authors’ conclusions regarding the overall reduction in symptoms and safety profile to estimate efficacy. In our literature review, all studies reported a reduction in aggression through standardized observations after IV-VPA therapy. Only in one case (involving two out of five patients, aged between 12 and 18), Thakur et al. (2004) [68] found that IV-VPA therapy was discontinued due to serious side effects such as gastrointestinal and neurological issues such as excessive sedation or bizarre behaviors. However, they concluded that IV valproate loading was still useful and safe. All other studies did not find adverse effects severe enough to warrant discontinuing treatment, compared to the benefits received. The efficacy of IV-VPA treatment in children and adolescents is summarized in Table 3 through coding papers in terms of yes, no, or no differences (see the legend: 1 = yes; 0 = no; X = no differences in Table 3 below).

Fontana et al. [58] mentioned only two papers that used IV-VPA in children and adolescents during an acute manic phase of bipolar disorder. These two studies were those of Thakur et al. (2004) [68] and Shah et al. (2003) [69], who respectively reported two patients aged 16 and 17 years, and five patients aged between 12 and 18 years. These two articles were not considered by Tripodi et al. (2023) [4], who instead mentioned a total of seven pediatric patients who underwent therapy with IV-VPA for the management of psychomotor agitation, who were included in the series of clinical cases reported by Battaglia et al. [28] and Hilthy et al. [29], but not by Fontana et al. (2019) [58]. Our research, therefore, updates the total number of studies on the use of IV-VPA in pediatric patients from the four mentioned by Tripodi et al. [4] to six studies in total, excluding our review. Furthermore, the age of children and adolescents treated with IV-VPA for agitation ranges from 7 to 14, excluding our case report. Overall, immediate relief from acute aggressive agitation symptoms with IV-VPA was reported in all studies included.

The reductions in agitation symptoms and aggressive behaviors were measured through direct observations using the Modified Overt Aggression Scale, the Brief Psychiatric Rating Scale, and the Children’s Global Assessment Scale in the six case reports of Battaglia C. et al. [28]. The Overt Aggression Scale was used for measurement by clinicians in only one study describing a patient with aggressive behaviors and agitation with ASD, Hilty et al. [29]. Finally, Fontana et al. [58] reported two articles describing a total number of seven pediatric patients: the five reported by Thakur et al. (2004) [68] were evaluated before and after IV-VPA using The Scale for Mania and Mixed Affective States; meanwhile, the two adolescents reported in the study of Shah et al. (2003) [69] were observed daily for maniac symptoms of agitation and for the presence/absence of side effects. In the latter article [69], the authors did not refer to any standardized instrument; however, they methodologically observed their patients every day with a specialized visit, registering each adverse effect and a reduction in symptom severity (see Table 3 and Table 4). Different dosages were used, depending on the stage of the acute episode. In the hyperacute stage, the induction dose was 1400 mg per day (15–20 mg/kg). The duration of the first phase ranged between 10 min (single IV dose) and 5 days. All reports described a gradual dose reduction within a range of 3 to 17 days. After tapering off IV-VPA, oral VPA was prescribed for up to six months or until complete clinical remission. This was done to avoid the risk of developing a rebound in aggressiveness or psychomotor effects caused by abrupt discontinuation, as has been observed in children treated for headaches [70] and as previously conducted by Battaglia et al. (2018) [28]. In these clinical cases, oral VPA doses were maintained within the range of 355 to 1000 mg per day. The blood concentration of VPA for each patient was monitored (50–100 μg/mL), and dosage adjustments were made to maintain the blood levels of ammonia (19–54 μg/mL) and liver enzymes (AST < 34 U/L; ALT 10–49 U/L; GGT < 73 U/L) within acceptable parameters. Consequently, the VPA dosage was adjusted based on the clinical response to symptoms, such as aggressive behavior and maniac/mixed symptoms [5].

All patients received concomitant medication, either initiated prior to the onset of acute agitation or as an add-on therapy.

The most commonly prescribed medications were anticholinergic drugs, benzodiazepines, first-generation antipsychotics, mood stabilizers, and second-generation antipsychotics. The most commonly used tools for measuring a reduction in aggressive behaviors included the Modified Overt Aggression Scale, the Overt Aggression Scale, the Aberrant Behavior Checklist, the Brief Psychiatric Rating Scale, the Bech–Rafaelsen Mania Scale, and the Clinical Global Impression. In some cases, when a psychiatric evaluation was possible, the authors mentioned the Mini-Mental State Examination.

In conclusion, a significant reduction in symptoms can be observed when IV-VPA is used to treat resistant agitation with aggressive behaviors in children and adolescents, as demonstrated by various scales and interviews, and the majority of reports exhibited an improvement with acute treatment of these patients. The safety profile was satisfactory, with only mild to moderate sedation and no significant alteration of blood analyses. The most common side effects of IV valproate include dizziness, headache, nausea, somnolence, and vomiting; these occurred in less than two of the patients to a severe degree (14.28%; n = 14 patients). In the other cases, the pediatric patients presented such effects in a transient and mild manner (see Table 4).

Finally, despite the heavy focus on comorbid ASD and ADHD in this study, as reflected by their explicit inclusion in the search criteria, no article analyzing the issue of IV-VPA utilization in people with these co-occurring clinical manifestations could be found, to the best of our knowledge.

## 5. Discussion

Treating pediatric patients with acute agitation and comorbid neurodevelopmental conditions such as ASD and ADHD is one of the most challenging tasks for clinicians [3,5,6]. Most severe agitation events are treated with neuroleptics, although, for those with complex neurodevelopmental disorders, they should be started at lower doses and with slower adjustment compared to other patients [3]. Furthermore, close monitoring of the total daily dose is recommended. Oral second-generation antipsychotics (e.g., risperidone, olanzapine, aripiprazole, and ziprasidone) along with the first-generation antipsychotics (e.g., haloperidol), whether administered as monotherapy or in combination with benzodiazepines, have demonstrated comparable effectiveness to oral VPA.

Our case report and literature review both indicated that, compared to oral VPA [24,25,26], the intravenous use of VPA in addition to neuroleptics in critical patients between 8 and 17 years is rapidly effective [4], even in the presence of ASD comorbidity [4,16].

In terms of safety, as our study was focused on the developmental age, we can report on the dosages used in the existing literature, presented and reported in children and adolescents aged between 8 and 17 years, regarding the use of IV-VPA in acute cases as a second-choice option when first-line treatments do not help reduce symptoms of aggression and agitation. Furthermore, in all of the studies considered, no damage to organic function was reported following the administration of IV-VPA for a limited time—namely, until the disappearance of acute symptoms of agitation and aggression—in minors under 18 years old. However, to date, there have been no clinical research studies evaluating its potential long-term adverse effects. In conclusion, given that psychomotor agitation and aggressive behaviors are among the most common reasons for which children and adolescents with ASD and ADHD are hospitalized urgently, we also specify that further RCT studies are necessary to evaluate long-term follow-up through larger case studies addressing pharmacogenomic/genetic issues related to the use of IV-VPA.

The intravenous administration of VPA in children and adolescents is currently limited to those who do not respond to other treatments and those who do not follow treatment plans. This restriction mainly exists as most studies on VPA have focused on adults with bipolar disorder. However, based on our analysis of the related literature and our own case studies, we believe that IV-VPA could be an effective and safe treatment for acute agitation and aggressive behavior in children and adolescents with neurodevelopmental disorders such as ASD and ADHD. Additionally, the safety profile of IV-VPA suggests that it could be a preferred option, even in cases where patients have a history of substance abuse and are experiencing agitation [28].

Furthermore, despite the focus on the comorbidity between ASD and ADHD in our study being explicitly included in the search criteria, we did not find any articles analyzing the use of IV-VPA for emergency psychiatric use in pediatric individuals with ASD and ADHD. Therefore, further research addressing these issues is necessary, given the high prevalence of this comorbidity and the needs of individuals with these conditions. Further exploration of the use of IV-VPA in this population can enhance our understanding of its therapeutic potential effects, and may assist clinicians in making therapeutic decisions during emergency management for acute agitation and aggressiveness in individuals who do not respond to first-line treatments. In the literature, stressor events continue to promote acute symptoms in patients with previous diagnoses of neurodevelopmental disorders; accordingly, our case was exposed to a traumatic event several months before the onset of the acute phase. This underscores the importance of considering such occurrences to mitigate environmental factors that could precipitate aggressive behaviors and agitation in susceptible children.

## 6. Conclusions

Our case is the first clinical case on the empirical use of IV-VPA for acute agitation and aggressive behaviors in a male child with co-occurring ASD and ADHD. The intravenous administration of valproic acid (VPA) was effective in reducing symptoms of agitation and immediate aggressiveness shortly after the infusion began, as a loading dose, within the first 30 min. This helped the child to return to his normal daily routine, continue with ongoing psychotherapy, and eventually resume school after a period of psychotic decompensation and aggressiveness that had led to a complete disruption of activities, necessitating a visit to the emergency room. Although VPA did not have a specific effect on reducing the core symptoms of ASD or ADHD, it was successful in decreasing acute aggressive behaviors related to psychomotor agitation, which is common in children and adolescents with complex neurodevelopmental disorders, as presented in this clinical case. Additionally, VPA was well tolerated by the patient and proved to be more effective than high-dose antipsychotic medications. The patient currently requires medium to high levels of support for autism spectrum disorder and has experienced a decrease in the severity of ADHD symptoms from severe to moderate. Therapy with methylphenidate, in combination with executive function training, has been effective in reducing the patient’s inattentiveness and distractibility with good safety and tolerance. Only one mild episode of decompensation—not requiring hospitalization—occurred seven months after the previous episode, possibly due to low levels of lithium sulfate in the blood caused by the child’s resistance to taking medication. Environmental and family issues reported by the mother also contributed to increasing the child’s stress levels in the same period as the relapse.

Moreover, our literature review updates the total number of studies on the use of IV-VPA in pediatric patients, from the previous four up to seven studies, including the present work, accounting for a total number of 15 children and adolescents treated with IV-VPA in the emergency psychiatry context (including our case report), compared to the previous seven patients mentioned in [4].

Further studies are needed to evaluate whether intravenous valproic acid (IV-VPA) could be a first-line therapeutic option for the treatment of acute and drug-resistant agitation in children with autism spectrum disorder (ASD) and attention-deficit/hyperactivity disorder (ADHD). Additionally, lifestyle interventions other than medication and pharmacological therapy could be examined in order to assess their effectiveness in reducing the number of recurrences of aggressive behaviors and psychomotor agitation experienced by these children. Although there exists an awareness of the benefits that activities such as meditation, healthy eating, exercise, reading, playing, and art activities have on the quality of life of individuals in general and children with neurodevelopmental disorders in particular, there is a lack of specific scientific literature evaluating the effectiveness of these interventions compared to pharmacological studies. This may be related to difficulties in standardizing the efficacy, reliability, and replicability of non-pharmacological treatment methods for ASD and associated neurodevelopmental disorders. The importance of having validated non-pharmacological treatment options is undoubtedly a challenge for clinical research in this field, as it has been established that various mechanisms involving genetic predisposition, environmental factors, and lifestyle play a role in ASD and ADHD, contributing significantly to variations in the trajectory of brain development. Therefore, future research should also analyze the effectiveness of interventions that can significantly influence the lifestyles of individuals with neurodevelopmental disorders, in addition to pharmacological trials. Finally, the latest national guidelines for ASD *(*https://www.iss.it/documents/20126/8977108/Linea+Guida+ASD_bambini+e+adolescenti+2023.pdf/e370f693-d569-4490-6d51-8e249cd152b0?t=1696841617387 (accessed on 9 October 2023)) do not recommend treating patients with ASD with methylphenidate (MPH), except for those with comorbid ADHD leading to high levels of hyperactivity, impulsivity symptoms, and attention deficits that clinically impact the individual’s life. Regarding the use of MPH for the treatment of ADHD, there is abundant evidence in the existing literature demonstrating its high efficacy in controlling inattentive and hyperactive-impulsive symptoms in children with ADHD [14], with good safety and tolerance profiles over a 2-year period in relation to growth and development, psychiatric health, neurological health, and cardiovascular function in children and adolescents, as well as a lower presence of comorbidities and a lower risk of developing other psychiatric comorbidities in late adolescence and young adulthood [15].

In summary, our clinical case and our review of the recent studies suggest that IV-VPA could be a possible alternative treatment that reduces the risk of adverse events associated with high-dose standard medications in the context of emergency psychiatry. However, further studies are required to evaluate whether IV-VPA could be an effective and safe option for treating acute and drug-resistant agitation in children with complex neurodevelopmental disorders.

## Figures and Tables

**Table 1:** Blood and instrumental tests.

Exams (Blood Levels)	Range	2 March 23	4 March 23	6 March 23	8 March 23	10 March 23
Lithium	0.6–1.2 mEq/L	*	*	*	0.35	0.53
Ammonia	19–54 μg/mL	46	*	73	97	96
VPA	50–100 μg/mL	*	*	92.7	83.4	63.8
PRL	3.2–13.5 ng/mL	*	16.9	*	41.38	43.01
AST	<34 U/L	58	42	32	27	27
ALT	10–49 U/L	34	29	24	16	16
LDH	120–246 U/L	433	401	367	280	277
GGT	<73 U/L	18	*	19	17	18
TBIL	0.3–1.2 mg/dL	0.7	0.5	0.5	0.6	0.4
Cr	0.6–1.1 mg/dL	0.69	0.69	0.68	0.75	0.71
ECG						
QT_C_	<440 ms	422 ms	406 ms	439 ms	395 ms	397 ms

LEGEND: * not evaluated; VPA = valproic acid; PRL = prolactin; AST = aspartate transaminase; ALT = alanine aminotransferase; LDH = lactate dehydrogenase; GGT = gamma-glutamyl transferase; TBIL = total bilirubin; Cr = creatinine; and QTc = correct QT interval.

**Table 2:** Screened articles on the use of VPA to treat acute agitation.

Title	Author (s)	Link	Study	Previous Diagnosis	Age	Administration
Effectiveness and safety of intravenous valproate in agitation: a systematic review	Olivola et al. (2022) [16]	https://link.springer.com/article/10.1007/s00213-021-06009-0 (accessed on 12 June 2024)	SR	AD; ADHD; ASD; BD; CD; MD; MDD; ODD; PSY; PTSD; SA; Schizoph.	C; Ado; Adu	IV
2. Efficacy and safety of valproic acid versus haloperidol in patients with acute agitation: results of a randomized, double-blind, parallel-group trial	Asadollahi et al. (2015) [45]	https://journals.lww.com/intclinpsychopharm/abstract/2015/05000/efficacy_and_safety_of_valproic_acid_versus.3.aspx (accessed on 12 June 2024)	RCT	AJ; MD; PSY	Adu	IV
3. Efficacy of topiramate, valproate, and their combination on aggression/agitation behavior in patients with psychosis	Gobbi G. et al. (2006) [46]	https://journals.lww.com/psychopharmacology/abstract/2006/10000/efficacy_of_topiramate,_valproate,_and_their.5.aspx (accessed on 12 June 2024)	CCS	BD; SAD; Schizoph.	Adu	O
4. Is anticonvulsant treatment of mania a class effect? Data from randomized clinical trials.	Rosa AR et al. (2011) [47]	https://onlinelibrary.wiley.com/doi/10.1111/j.1755-5949.2009.00089.x (accessed on 12 June 2024)	SR	Mania	NA	O
5. Significant Effect of Valproate Augmentation Therapy in Patients With Schizophrenia: A Meta-analysis Study	Tseng PT et al. (2016) [48]	https://journals.lww.com/md-journal/fulltext/2016/01250/significant_effect_of_valproate_augmentation.12.aspx (accessed on 12 June 2024)	MA	SAD; Schizoph.	NA	O
6. Valproate or olanzapine add-on to lithium: an 8-week, randomized, open-label study in Italian patients with a manic relapse	Maina G et al. (2007) [49]	https://www.sciencedirect.com/science/article/pii/S0165032706003922?via%3Dihub (accessed on 12 June 2024)	RCT	BD	Adu	O
7. Intravenous valproate in neuropsychiatry	Norton JW and Quarles E. (2000) [50]	https://accpjournals.onlinelibrary.wiley.com/doi/10.1592/phco.20.1.88.34657 (accessed on 12 June 2024)	SR	BD; Mania	Adu	IV
8. Lessons learned from the discovery of sodium valproate and what has this meant to future drug discovery efforts?	Janković SM and Janković SV. (2020) [17]	https://www.tandfonline.com/doi/full/10.1080/17460441.2020.1795125 (accessed on 12 June 2024)	R	BD	C; Ado	O
9. A rapid and systematic review and economic evaluation of the clinical and cost-effectiveness of newer drugs for treatment of mania associated with bipolar affective disorder	Bridle C et al. (2004) [21]	https://www.journalslibrary.nihr.ac.uk/hta/hta8190/#/abstract (accessed on 12 June 2024)	R	BD	C; Ado; Adu	O
10. Long-Term Treatment of Bipolar Disorder with Valproate: Updated Systematic Review and Meta-analyses	Yee CS et al. (2021) [22]	https://journals.lww.com/hrpjournal/abstract/2021/05000/long_term_treatment_of_bipolar_disorder_with.2.aspx (accessed on 12 June 2024)	R; MA	BD	Adu	O
11. A systematic review and economic model of the clinical effectiveness and cost-effectiveness of interventions for preventing relapse in people with bipolar disorder	Soares-Weiser K et al. (2007) [51]	https://www.journalslibrary.nihr.ac.uk/hta/hta11390/#/abstract (accessed on 12 June 2024)	SR	BD	Adu	O
12. Changes in body weight and body mass index among psychiatric patients receiving lithium, valproate, or topiramate: an open-label, nonrandomized chart review	Chengappa KN et al. (2002) [52]	https://www.clinicaltherapeutics.com/article/S0149-2918(02)80061-3/abstract (accessed on 12 June 2024)	R	BD; SA; SAD; MDD; Schizoph.	NA	NA
13. Pharmacokinetics, drug interactions, and tolerability of valproate	DeVane CL (2003) [53]	https://pubmed.ncbi.nlm.nih.gov/14624231/(accessed on 13 June 2024)	R	BD	C; Ado; Adu	O
14. Valproate in the treatment of PTSD: systematic review and meta analysis	Adamou M et al. (2007) [54]	https://www.tandfonline.com/doi/abs/10.1185/030079907X188116 (accessed on 12 June 2024)	SR, MA	PTSD	Adu	O
15. Valproate for agitation in critically ill patients: A retrospective study	Gagnon DJ et al. (2017) [55]	https://www.sciencedirect.com/science/article/pii/S0883944116301964?via%3Dihub (accessed on 12 June 2024)	RS	AD; ADHD; BD; MDD; PTSD	Adu	O
16. Valproate as an adjunct to antipsychotics for schizophrenia: a systematic review of randomized trials	Basan A et al. (2004) [56]	https://www.sciencedirect.com/science/article/pii/S0920996404000477?via%3Dihub (accessed on 12 June 2024)	SR	SAD; Schizoph.	NA	O
17. The emerging role of valproate in bipolar disorder and other psychiatric disorders	Guay DR (1995) [57]	https://accpjournals.onlinelibrary.wiley.com/doi/10.1002/j.1875-9114.1995.tb02874.x (accessed on 12 June 2024)	R	BD; SAD	C; Ado; Adu	O
18. Intravenous Valproic Acid Add-On Therapy in Acute Agitation Adolescents With Suspected Substance Abuse: A Report of Six Cases	Battaglia C et al. (2018) [28]	https://journals.lww.com/clinicalneuropharm/abstract/2018/01000/intravenous_valproic_acid_add_on_therapy_in_acute.9.aspx (accessed on 12 June 2024)	CR	CD; MD; ODD; PSY; SA	Ado	IV
19. Intravenous valproate in the treatment of acute manic episode in bipolar disorder: A review	Fontana E (2019) [58]	https://www.sciencedirect.com/science/article/pii/S0165032719315903?via%3Dihub (accessed on 12 June 2024)	R	BD	C; Ado; Adu	IV
20. Sodium valproate for the treatment of Tourette’s syndrome in children: a systematic review and meta-analysis	Yang CS et al. (2015) [59]	https://www.sciencedirect.com/science/article/pii/S0165178114007550?via%3Dihub (accessed on 12 June 2024)	SR, MA	TS	C; Ado	O
21. Valproate for schizophrenia	Schwarz C et al. (2008) [60]	https://www.cochranelibrary.com/cdsr/doi/10.1002/14651858.CD004028.pub3/full (accessed on 12 June 2024)	R	PSY; Schizoph.	Adu	O
22. Safety and tolerability of mood-stabilising anticonvulsants in the elderly	Fenn HH et al. (2006) [61]	https://www.tandfonline.com/doi/full/10.1517/14740338.5.3.401 (accessed on 12 June 2024)	R	MD	Adu	O
23. Combination of Olanzapine and Samidorphan Has No Clinically Significant Effect on the Pharmacokinetics of Lithium or Valproate	Sun L et al. (2019) [62]	https://link.springer.com/article/10.1007/s40261-019-00860-y (accessed on 12 June 2024)	CS	BD	Adu	O
24. Valproic acid, valproate and divalproex in the maintenance treatment of bipolar disorder	Cipriani A et al. (2013) [23]	https://www.cochranelibrary.com/cdsr/doi/10.1002/14651858.CD003196.pub2/full (accessed on 12 June 2024)	R	BD	C; Ado; Adu	O
25. Valproate as an adjunct to neuroleptic medication for the treatment of acute episodes of mania: a prospective, randomized, double-blind, placebo-controlled, multicenter study European Valproate Mania Study Group.	Müller-Oerlinghausen B et al. (2000) [63]	https://journals.lww.com/psychopharmacology/abstract/2000/04000/valproate_as_an_adjunct_to_neuroleptic_medication.12.aspx (accessed on 12 June 2024)	CT	Mania	Adu	O
26. The emerging story of Sodium Valproate in British newspapers- A qualitative analysis of newspaper reporting	Siriwardena S et al. (2022) [64]	https://www.seizure-journal.com/article/S1059-1311(22)00172-8/fulltext (accessed on 12 June 2024)	TA	SAD	C; Ado; Adu	NA
27. Adjunctive valproic acid for delirium and/or agitation on a consultation-liaison service: a report of six cases	Bourgeois JA et al. (2005) [65]	https://neuro.psychiatryonline.org/doi/full/10.1176/jnp.17.2.232 (accessed on 12 June 2024)	CR	A, BD,PTSD, Schizoph.	Adu	IV
28. Intravenous valproate for rapid stabilization of agitation in neuropsychiatric disorders	Hilty DM et al. (1998) [29]	https://neuro.psychiatryonline.org/doi/full/10.1176/jnp.10.3.365 (accessed on 12 June 2024)	CR	ASD	C	IV
29. Valproic acid for treatment of hyperactive or mixed delirium: rationale and literature review	Sher Y et al. (2015) [66]	https://www.sciencedirect.com/science/article/pii/S0033318215001577?via%3Dihub (accessed on 12 June 2024)	R	Delirium	Ado; Adu	IV
30. A Critical Review of the Psychomotor Agitation Treatment in Youth	Tripodi B et al. (2023) [4]	https://www.mdpi.com/2075-1729/13/2/293 (accessed on 12 June 2024)	R	AD;ADHD; ASD; BD; CD; MD; ODD; PSY; PTSD, SA	C; Ado; Adu	IV
31. A double-blind, placebo-controlled study of valproate for aggression in youth with pervasive developmental disorders	Hellings J.A. et al. (2005) [24]	https://www.liebertpub.com/doi/10.1089/cap.2005.15.682 (accessed on 12 June 2024)	RCT	ASD	C; Ado; Adu	O
32. Divalproex sodium vs. placebo in the treatment of repetitive behaviors in autism spectrum disorder	Hollander E. et al. (2006) [26]	https://academic.oup.com/ijnp/article/9/2/209/674335 (accessed on 12 June 2024)	CT	ASD	C; Ado; Adu	O
33. Divalproex sodium vs. placebo in the treatment of irritability in children and adolescent with autism spectrum disorder	Hollander E. et al. (2010) [25]	https://www.nature.com/articles/npp2009202 (accessed on 12 June 2024)	CT	ASD	C; Ado	O
34. Valproate for schizophrenia (Review)	Wang Y. et al. (2016) [67]	https://www.cochranelibrary.com/cdsr/doi/10.1002/14651858.CD004028.pub4/full (accessed on 12 June 2024)	R	SAD; Schizoph.	Adu	NA

LEGEND: study: CR = case report; CS = cohort study; CCS = case control study; CT = clinical trial; MA = meta-analysis; PS = comparative study; SR = systematic review; R = literature review; RCT = randomized clinical trial; TA = thematic analysis; Diagnosis: A = agitation; AD = anxiety disorder; ADHD = attention-deficit/hyperactive disorder; AJ = adjustment disorder; ASD = autism spectrum disorder; BD = bipolar disorder; CD = conduct disorder; MD = mood disorder; ODD = oppositional defiant disorder; Ot = others; PSY = psychosis; SA = substance abuse; SAD = schizoaffective disorder; Schizoph = schizophrenia; age: C = children (<13 year); Ado = adolescent (13 < Age < 18); Adu = adult (>18 year); NA = not specified; administration: IV = intravenous; O = oral; NA = not specified.

**Table 3:** Response to IV-VPA in children and adolescents with acute aggressive agitation.

Study	Link	Study Design	Diagnosis	Administration	DOSAGE/PHASES	Period of Treatment	Concomitant Medication	Outcomes	Response
Intravenous Valproic Acid Add-On Therapy in Acute Agitation Adolescents with Suspected Substance Abuse: A Report of Six Cases. (Battaglia C et al., 2018) [28]	https://journals.lww.com/clinicalneuropharm/abstract/2018/01000/intravenous_valproic_acid_add_on_therapy_in_acute.9.aspx	Case report	CD; MD; ODD; PSY; SA	IV	1200–1800 mg/day	5–17 days	SGAs MS BDZ	MOAS BPRS	1
2. Intravenous valproate in the treatment of acute manic episode in bipolar disorder: A review (Fontana E 2019) [58]	https://www.sciencedirect.com/science/article/pii/S0165032719315903?via%3Dihub	Review	BD	IV	800–1500 mg/day 250 mg from one to three times a day	3 days 15 days	FGAs, BDZ SGAs	BRMAS YMRS CGI-S MMSE	1
3. Intravenous valproate for rapid stabilization of agitation in neuropsychiatric disorders (Hilty DM et al., 1998) [29]	https://neuro.psychiatryonline.org/doi/full/10.1176/jnp.10.3.365	Case report	ASD	IV O	Loading phase: 2000 mg/day (40 mg/kg) Maintenance dose: 1000 mg/day	10′ 6 months	SGAs, ACH	OAS	1
4. A Critical Review of the Psychomotor Agitation Treatment in Youth (Tripodi B et al., 2023) [4]	https://www.mdpi.com/2075-1729/13/2/293	Review	ASD; CD; MD; ODD; PSY; SA	O IV IV	950 ± 355 mg 1200–1800 mg/day 2000 mg/day (40 mg/kg)	6 months 5–17 days 10′	NA	/	1

LEGEND: Diagnosis: AD = anxiety disorder; ADHD = attention-deficit/hyperactive disorder; ASD = autism spectrum disorder; BD = bipolar disorder; CD= conduct disorder; MD = mood disorder; ODD = oppositional defiant disorder; PSY = psychosis SA = substance abuse; Schizophr. = schizophrenia; administration: IV = intravenous; O = oral; concomitant medication: ACH = anticholinergic; BDZ = benzodiazepines; FGAs = first-generation antipsychotics; MS = mood stabilizer; SGAs = second-generation antipsychotics; outcomes: ABC = Aberrant Behavior Checklist [31]; BPRS = brief psychiatric rating scale [32]; BRMAS = Bech–Rafaelsen Mania Scale [33]; CGI= clinical global impression [34]; CY-BOCS = Children’s Yale–Brown Obsessive Compulsive Scale [35]; MMSE = mini mental state examination [36]; MOAS = Modified Overt Aggression Scale [38]; OAS = Overt Aggression Scale [37].

**Table 4:** Symptom modifications and Side Effects of administered medications in add-on therapies during IV-VPA administration Period.

Patient ID	Authors	Age	Sex	Add-on Therapy During IV-VPA Administration Period	Symptom Modifications (* = Mild; ** = Moderate; *** = Marked)	Side Effects
1	Hilthy et al., 1998 [29]	8 years	Female	None	Marked symptom reduction in 15′ after IV-VPA started ***	None
2	Shah et al., 2003 [69]	17 years	Female	None	Reduction in mania behaviors ***	One episode of nausea and vomiting
3	Shah et al., 2003 [69]	16 years	Female	None	No improvement in symptoms was observed after the infusion on day 1, and even on day 5, there was only a minimal improvement.	On day 6, patient developed giddiness, ataxia, and incoordination; therefore, the therapy was discontinued
4	Thakur et al., 2004 [68]	15 years	Male	Haloperidol 15 mg/day (it starts after two days of IV-VPA for persisting paranoia)	Reduction in aggressive *** behaviors; no reduction in mania symptoms	None
5	Thakur et al., 2004 [68]	14 years	Male	Lorazepam (4 mg) single administration during the 1st day	Reduction in psychomotor agitation **	Transient headache, dizziness, and nausea from the second day
6	Thakur et al., 2004 [68]	15 years	Male	None	Reduction in aggressive behaviors ***	None
7	Thakur et al., 2004 [68]	15 years	Male	None	Reduction in psychomotor agitation ***	Transient headache, tingling in hands and feet, and gastrointestinal side effects
8	Thakur et al., 2004 [68]	15 years	Male	Lorazepam (4 mg) on the first day.	Reduction in psychomotor agitation ** and aggressive behaviors ***	None
9	Battaglia et al., 2018 [28]	17 years	Female	Clotiapine 99 mg Diazepam 8 mg Lorazepam 4 mg im	Reduction in manic/mixed symptoms ***	Hair loss
10	Battaglia et al., 2018 [28]	16 years	Male	Clopromazine 1 fl im Promazine 1 fl im diazepam 1 fl im	Reduction in aggressive behaviors ***	None
11	Battaglia et al., 2018 [28]	17 years	Male	Lorazepam 4 mg VPA 500 mg	Reduction in aggressive behaviors ***	None
12	Battaglia et al., 2018 [28]	17 years	Male	Diazepam 5 mg	Reduction in manic/mixed symptoms ***	None
13	Battaglia et al., 2018 [28]	16 years	Male	Clonazepam 2.5 mg Olanzapina 10 mg VPA 500 mg Lorazepam 4 mg iv	Reduction in aggressive behaviors ***	None
14	Battaglia et al., 2018 [28]	17 years	Male	Clopromazine 50 mg im Clotiapine 66 mg	Reduction in aggressive behaviors *** and anxious symptoms ***	Skin rash

LEGEND: The Table 4 shows that side effects of administered medications in add-on therapies during IV-VPA administration period, was severe just in two cases: one in the 15 years old [69] and one in a 17 years old [28] girls. The first case showed giddiness, ataxia, and incoordination [69] side effects and in the second case hair loss [28] was observed. Just in one of these two clinical cases, the side effect was related to the IV VPA, as the patient was not taking other medications in add-on therapy and treatement was discontinued, also due to the lack of improvement in maniac symptoms [69]. In all the other cases reduction in psychomotor agitation, aggressive behaviors or in manic/mixed symptoms, were obtained.

## Data Availability

The data presented in this study are available on request from the corresponding author due to replicability in this research field.
